# Water supply emergency preparedness and response in health care facilities: A systematic review on international evidence

**DOI:** 10.3389/fpubh.2022.1035212

**Published:** 2022-12-05

**Authors:** Sophie van der Heijden, Alexandra Cassivi, Aljoscha Mayer, Simone Sandholz

**Affiliations:** ^1^United Nations University - Institute for Environment and Human Security (UNU-EHS), Bonn, Germany; ^2^Chaire de recherche en eau potable, École supérieure d'aménagement du territoire et de développement regional, Université Laval, Québec, QC, Canada

**Keywords:** water supply and wastewater management, health care facilities, emergency preparedness, disaster response, risk assessment, low-resource contexts

## Abstract

**Introduction:**

Enabling health care facilities to deal with impairments or outages of water supply and sewage systems is essential and particularly important in the face of growing risk levels due to climate change and natural hazards. Yet, comprehensive assessments of the existing preparedness and response measures, both in theory and practice, are lacking. The objective of this review is to assess water supply and wastewater management in health care facilities in emergency settings and low-resource contexts. It thereby is a first step toward knowledge transfer across different world regions and/or contexts.

**Method:**

A systematic review was performed to identify published articles on the subject using online MEDLINE and Web of Science. The initial searches yielded a total of 1,845 records. Two independent reviewers screened identified records using selection criteria. A total of 39 relevant studies were identified. Descriptive analyses were used to summarize evidence of included studies.

**Results:**

Overall, water supply was far more discussed than wastewater management. Studies on emergency preparedness identified back-up water storage tank, additional pipelines, and underground wells as key sources to supply health care facilities with water during an emergency. In emergency response, bottled of water, followed by *in-situ* back-up water storage tanks previously installed as part of disaster preparedness measures, and tanker trucks to complete were most used. Questions on how to improve existing technologies, their uptake, but also the supplementation by alternative measures remain unanswered. Only few guidelines and tools on emergency preparedness were identified, while multiple studies formulated theoretical recommendations to guide preparedness. Recovery planning was rarely discussed, despite many studies mentioning the importance of the reconstruction and restoration phases. Literature focus on recovery is mostly on technical aspects, while organizational ones are largely absent. Despite their key role for preparedness and response, citizens and patients' perspectives are hugely underrepresented. This fits into the bigger picture as communication, awareness raising and actor cooperation in general is addressed comparatively little.

**Discussion:**

Combining organizational and technical aspects, and intersecting theory and practice will be necessary to address existing gaps. Improving both, preparedness and response, is key to maintaining public health and providing primary care.

## Introduction

The provision of basic water, sanitation and hygiene (WASH) services in health care facilities (HCF) is essential for maintaining public health and providing primary care, i.e., safe and accessible water supply, on-site sanitation, hygiene facilities and waste management. Despite making WASH a core priority under Sustainable Development Goal No. 6 and significant progress in the last decades, in 2021, more than one out of five health care facilities worldwide had no basic water services (1). With significant impacts on infrastructure and provision of services, lack of access to WASH can contribute to the spread of diseases and increase health care-associated infection within HCF and surroundings (2). This is especially true for the most vulnerable populations and environments, e.g., marginalized and economically disadvantaged groups, humanitarian emergencies and crisis settings, post-disaster shelter, refugee camps, which are disproportionally affected by lack of basic services (3).

In a context of rising climate uncertainties and emerging threats, WASH and health infrastructure are subject to ever-increasing fragilities (4). Human and environmental hazards, e.g., climate or extreme weather events, armed conflict and terrorist attacks, and water supply failures or disruptions (e.g., due to power outages, dam failure, chemical spill), increase risk and may compromise the reliability of basic services. The COVID-19 pandemic is a stark reminder of ongoing global challenges and adverse effects on critical health infrastructure, e.g., deficiencies of health care facilities due to lack of preparedness (5).

Effective water management and disposal strategies, in normal times and crisis events, are essential to enable health infrastructures to deliver health services (6, 7). Level of supply reliability and standards can significantly affect needs in case of service disruption. Low-awareness and high dependency to continued services are considered determinant factors of fragility (8). For example, in settings with a continuous water supply, higher quantities of water will be expected to reach limited level of service and cope with intermittency.

To minimize the impact and avoid cascading failures of other critical systems, water supply utilities and health care facilities must withstand by themselves and ensure that basic services are provided and can recover in the case of emergency events and disasters (9). Substantial efforts and investments toward basic needs assessment, emergency preparedness and response are required worldwide, and this is necessary to bridge existing gaps between emergency and development (10). Although crisis events generally capture more attention, improving strategic planning at all stages from prevention and mitigation to long-term recovery is primordial. Despite learning from devastating impacts, inadequate application and implementation of emergency preparedness and capacity response assessment persist (11). The challenges that relate to emergency water supply and treatment in health care facilities are frequently overlooked and under-documented.

This systematic review focuses on the provision of water and sanitation services and health care facilities in emergency settings and low-resource contexts. While emergency settings can be defined as an unexpected, especially dangerous situation which threatens human, material, economic or environmental assets, we focus in particular on emergency settings which threaten the water supply or waste water management of health care facilities. One example is last year's flood in the states of Rhineland-Palatinate and North Rhine-Westphalia, which particularly affected the water supply of flood-affected health care facilities. Similarly, as low-resource contexts is an umbrella term that indicates a deficiency in a variety of areas, we specifically looked at settings where the water supply was unreliable. An example might be the northern remote communities in Canada where water is supplied with truck-to-cistern. Both of these contexts can occur in high-income countries as well as in middle- and low-income countries.

The objective of this systematic review is to provide an overview of the existing preparedness and response measures and strategies that have been theorized and/or implemented to enable health care facilities to access water supply and wastewater management to maintain their operations in the event of a disruption or impairment. The specific objectives are twofold: (1) to examine existing international standards and guidelines for emergency water supply preparedness in health care facilities, and (2) to identify preparedness and response strategies, technical and organizational interventions and recovery plans for the provision of water supply and waste water management. By reviewing international evidence and providing an overview on the range of approaches to deal with past emergencies but also low-resource contexts this review closes a research gaps and is a first step toward knowledge transfer across different world regions and/or contexts. The review aims to answer the following question: What emergency preparedness and response measures and guidelines for water supply and wastewater management for health care facilities can be found through a systematic review of the scientific literature?

## Methods

This systematic review was conducted following the Preferred Reporting Items for Systematic reviews and Meta-Analyses (PRISMA) (12).

### Eligibility criteria

Studies that focus on water supply in health care facilities in emergency settings and low resource contexts were sought for inclusion in this study. The systematic review was conducted using only peer-reviewed literature searches. English was consistently used to yield searches. No restriction related to the publication language or date of coverage were, however, applied for the initial search.

### Information sources

A comprehensive literature search of peer-review publications was done through MEDLINE and Web of Science.

### Search strategy

The search strategy includes general sets of criteria related to water supply and health care facilities in low resource contexts including vulnerable and economically disadvantaged groups (e.g., low-and middle-income settings, Indigenous populations, refugee camps) as well as humanitarian emergencies and crisis settings. The peer-review literature was identified through databases tailored search using a combination of basic terms and subject terms that relates to the sets of criteria (i.e., context, water supply and waste water management, health care facilities, disaster planning and response) as well as controlled vocabulary search terms, including index or MeSh terms (e.g., Disaster, Water supply, Health facilities). The overall search strategy, including definitions of sets of criteria, is available as Supplementary material.

### Selection process

The selection process followed the PRISMA chart flow and guideline (13). The literature search was performed by one author, and records were retrieved in April 2022. The identified records were extracted using EndNote X9. Records published before 2000 and duplicates were removed before initial screening. The title and abstract of identified records were screened for eligibility by two independent reviewers (Heijden S. and Cassivi A.), and in case of any disagreement a third reviewer (Sandholz S.) was consulted for consensus. Full text of eligible studies, including peer-review articles, conference proceedings and reports, was assessed for eligibility using inclusion and exclusion criteria (Table 1). Records including reports and studies using empirical evidence that relates to water supply for health care facilities in low-resource contexts and/or emergency settings were sought for inclusion.

**Table 1:** Inclusion and exclusion criteria for selection of records (SPIDER).

	**Inclusion**	**Exclusion**
Sample	Settings/population: - Health care facilities, e.g., hospitals, clinics. - Low resource context, e.g., LMIC - Emergency, humanitarian settings	Settings/population: - Households or domestic settings - School or educational facilities - Veterinary or animal clinics
Phenomenon of interest	Research area: - Preparedness for water supply emergency and wastewater management - Provision of drinking water and other purposes. - Critical water uses in HCF - Evaluation of water supply - Response/Interventions/Mitigation measures/Solutions for water supply and wastewater management - Lessons learned from emergency water supply.	Research area: - Status of WASH coverage, conditions and compliance - Engineering technology and applied sciences - Epidemiological studies/ Public Health threats/ Health-associated infections - Hygiene practices/Environmental conditions - Water quality monitoring and environmental surveillance, e.g., Legionella contamination. - Pollution, e.g., pharmaceutiques components - Quality of health care service and satisfaction - Human resources and health care workers - Medical or medicine supply. - Financing mechanisms/Cost analysis - Models or simulations - Antibiotics resistance/residuals
Design	Any design	
Evaluation	Subjective or empirical evidence	
Research type	Qualitative, quantitative or mixed	

### Data extraction and synthesis

Data from included studies was extracted and compiled using a structured form (available on request). Extracted data included general descriptive and contextual information on water supply and health care facilities, actors and coordination efforts, as well as specific information on emergency preparedness, response and recovery. Studies were finally classified in general categories and analyzed using descriptive analysis and qualitative evidence synthesis. Due to the observed heterogeneity of selected evidence, no pre-defined tool for the assessment of quality of evidence was used. Synthesis from overall study assessment was included and quality assessment was reported independently when relevant.

## Results

### Selection of sources of evidence

**Figure 1:**
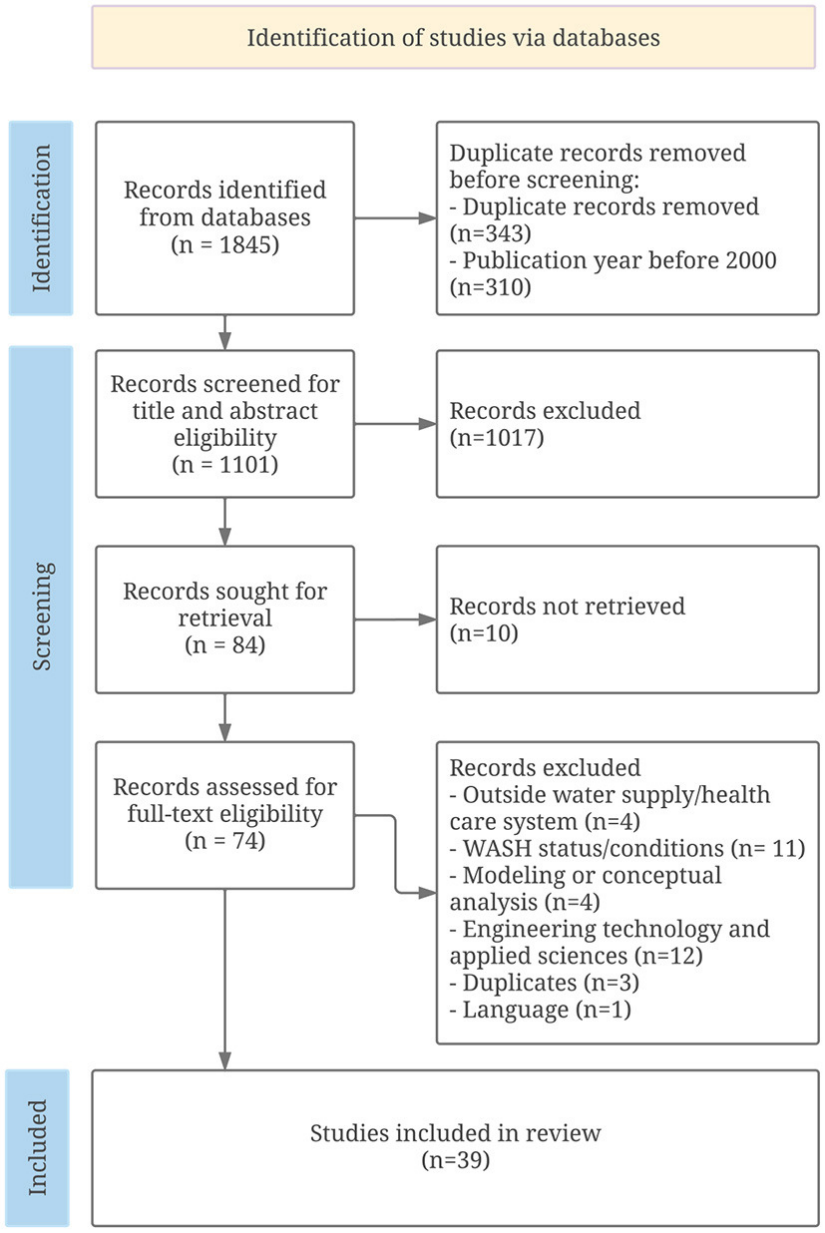
PRISMA Flow chart for study selection.

The initial search yielded 1,845 records. A total of 310 records were published before 2000 and 343 duplicates were initially excluded. Of the records eligible, 1,101 records were screened for title and abstract eligibility. Overall, 84 records were assessed for full text eligibility, of which 39 records were selected for inclusion in the systematic review. The search strategy and study selection are presented in the flow chart diagram (Figure 1).

### Summary of study characteristics

A prospective approach, through emergency preparedness and response capacity assessment, was used in forty percent of the studies while the remaining used a retrospective case report approach (Table 2). Overall, all (13 studies) but one study focusing on emergency preparedness were conducted in normal times, prior to an event of water supply and/or power supply emergency. Remaining studies, among which most are case disaster reports, were conducted in the event of a natural hazard-induced disaster (e.g., earthquake, flood, hurricane, COVID-19 pandemic). Case studies (23 studies) were conducted in a large range of different types of health care facilities, including hospitals (23 studies), disaster-based hospitals (4 studies), primary health care centers, and medical centers (13 studies), with a number of beds ranging from 18 to 1,000. Studies generally covered water supply and provision within health care facilities, and very few studies also discussed wastewater (5 studies) management. Finally, most studies included were conducted in high-income countries (25 studies), and the United States and Japan accounted for nearly half of the studies. Low- and middle-income countries (14 studies) were represented with individual studies from Cameroon, China, Haiti, Indonesia, Iran, Malaysia, Nepal, South Africa, Sri Lanka and Zambia. Two additional studies were, respectively, conducted worldwide and used a fictional country as a case study (Table 3).

**Table 2:** Classification of the selected studies (*N* = 39).

**Classification**	**Studies**
Disaster case report	59%
Risk assessment and preparedness	41%
High income countries	64%
Middle and low income countries	36%
Studies with implemented preparedness measures	26%
Studies with implemented response measures	46%

**Table 3:** General characteristics of included studies (*N* = 39).

**References**	**Type**	**Countries**	**Sample (Number HCF^a^, type^b^, number of beds^c^)**	**Emergency situation**	**Event**
Adhikari et al. (14)	Disaster case report	Nepal	NS, health facilities, NS	Natural hazard-induced disaster	Occurred
Alexakis et al. (15)	Risk assessment and preparedness	Greece	54 health facilities, 18 to 500 beds	Water and/or power supply emergency	Hypothetical
Ateudjieu et al. (16)	Risk assessment and preparedness	Cameroon	134 health facilities, NS	Health emergency (outbreak)	On going
Ballantyne (17)	Disaster case report	Sri Lanka	NS	Natural hazard-induced disaster	Occurred
Dippenaar (18)	Risk assessment and preparedness	South Africa	1 hospital, 334 beds	Water and/or power supply emergency	Hypothetical
Bichai et al. (19)	Risk assessment and preparedness	Worldwide	NA	Health emergency (outbreak)	Occurred
Bross et al. (7)	Risk assessment and preparedness	Germany and Austria	NS, health facilities in Germany (256 beds average) and Austria (239 beds average)	Water and/or power supply emergency	Hypothetical
Bross and Krause (20)	Risk assessment and preparedness	Germany	NS. hospitals, NS	Water and/or power supply emergency	Hypothetical
Cdc Environmental Health Services Branch (21)	Risk assessment and preparedness	USA	Case Study No. 1: 1 academic medical center, 700 beds Case Study No. 2: 1 Nursing home, 165 beds	Water and/or power supply emergency	Hypothetical
deBoisblanc (22)	Disaster case report	USA	1 hospital, 500 beds	Natural hazard-induced disaster	Occurred
Gray and Hebert (23)	Disaster case report	USA	dozen hospitals, NS	Natural hazard-induced disaster	Occurred
Haar et al. (24)	Disaster case report	Haiti	59 health care providers, NS	Natural hazard-induced disaster	Occurred
He et al. (25)	Disaster case report	China	1 temporary hospital, 1,000 beds case study	Health emergency (outbreak)	Occurred
Heidaranlu et al. (26)	Disaster case report	Iran	8 hospitals, 60 to 980 beds	Natural hazard-induced disaster	Occurred
Hsu et al. (27)	Disaster case report	USA	10 health facilities, 25 beds and more HCFs	Chemical emergency	Occurred
Ikegaya et al. (28)	Disaster case report	Japan	1 disaster base hospital, 712 beds	Natural hazard-induced disaster	Occurred
Inagaki and Sadohara (29)	Risk assessment and preparedness	Japan	134 disaster base hospitals, NS	Natural hazard-induced disaster	Hypothetical
Janius et al. (30)	Risk assessment and preparedness	Malaysia	5 government hospitals, NS	Water and/or power supply emergency	Hypothetical
Klein et al. (31)	Disaster case report	USA	4 hospitals (3 trauma centers and 1 children's hospital)	Power and water emergency	Occurred
Lapcevic et al. (32)	Risk assessment and preparedness	Serbia	1 hospital, 336 health care workers	Natural hazard-induced disaster	Occurred
Lestari et al. (33)	Risk assessment and preparedness	Indonesia	11 hospitals, NS	Water and/or power supply emergency	Hypothetical
Matsumura et al. (34)	Disaster case report	Japan	14 disaster base hospital, NS	Natural hazard-induced disaster	Occurred
Mitchell et al. (35)	Disaster case report	USA	1 hospital, NS	Natural hazard-induced disaster	Occurred
Nates (36)	Disaster case report	USA	1 medical center, 28 beds intensive care unit	Natural hazard-induced disaster	Occurred
Ochi et al. (37)	Disaster case report	Japan	147 hospitals, NS	Natural hazard-induced disaster	Occurred
Paez et al. (38)	Risk assessment and preparedness		NA	Water and/or power supply emergency	Hypothetical
Parmar et al. (39)	Disaster case report	Japan and USA	1 hospital, 778 beds	Natural hazard-induced disaster	Occurred
Perrin (40)	Disaster case report	USA	1 tertiary hospital, 54 beds	Natural hazard-induced disaster	Occurred
Redfern et al. (41)	Disaster case report	USA	1 tertiary hospital, 794 beds	Water and/or power supply emergency	Occurred
Roberson and Hiltebrand (42)	Risk assessment and preparedness	USA	NS, health facilities, NS	Water and/or power supply emergency	Hypothetical
Ryan et al. (43)	Disaster case report	Australia	No sample, public health infrastructure, NS	Natural hazard-induced disaster	Occurred
Salfarlie (44)	Risk assessment and preparedness	USA	1 hospital, NS	Water and/or power supply emergency	Occurred
Shimoto et al. (45)	Disaster case report	Japan	9 hospitals, NS	Natural hazard-induced disaster	Occurred
Sinyange et al. (46)	Disaster case report	Zambia	267 household	Health emergency (outbreak)	Occurred
Suginaka et al. (47)	Risk assessment and preparedness	Japan	1 university affiliate hospital, 653 beds	Natural hazard-induced disaster	Occurred
Wahren et al. (48)	Disaster case report	Poland and Sweden	NA (health care system)	Natural hazard-induced disaster	Occurred
Welter et al. (49)	Risk assessment and preparedness	USA	1 regional health center	Natural hazard-induced disaster	Occurred
WHO (50)	Disaster case report	Haiti	NS, health facilities, NS	Natural hazard-induced disaster	Occurred
Yusoff et al. (51)	Disaster case report	Malaysia and overseas	NS, hospitals, NS	Natural hazard-induced disaster	Occurred

^*a*^health care facilities; ^*b*^used term to describe the facility in which primary health care is provided (e.g., hospital, health center, clinic, dispensary etc.); ^*c*^not specified.

### Results of syntheses

Preparedness measures implemented in the event of water supply outages or impairments, existing guidelines and tools to prepare for this scenario, and written recommendations for improving preparedness (17 studies) were discussed in the literature. Many studies also address measures, tools and plans implemented as a response in the event of water supply outages or impairment (22 studies); and only a few look at the issue of recovery planning (5 studies).

#### Emergency preparedness

In most cases (17 studies from the 39 included), published literature on emergency water supply relates to risk assessment and emergency preparedness measures (Table 4). While some studies offered a general overview of preparedness measures, most studies performed an in-depth analysis of preparedness measures to prepare for a water impairment or outage. Examples of preparedness measures included implementation of emergency alternative water supply (e.g., emergency water tank) as well as emergency preparedness tools and plans for health care facilities (e.g., the hospital safety index). Among all studies, 11 studies that relate to emergency preparedness discussed the role of various stakeholders in facilitating networking and planning in the context of an emergency.

**Table 4:** Implemented preparedness measures for emergency water supply in health care facilities in the literature (*N* = 10/39).

**Implemented measures**	**Number of studies**	**References**
Water tanks	6	(15, 29, 34, 39, 40, 47)
Additional pipes	4	(21, 27, 28, 30)
Wells	3	(21, 28, 29)
No reference to implemented preparedness measures	29	All other papers from the literature review

##### Emergency alternative water supply

In the event of water impairment or outage, the provision of water supply is a priority to minimize the risk of service disruption. Overall, 10 studies discussed the implementation of emergency water supply preparedness measures in health care facilities (Table 4). Common measures included back-up water storage tanks, additional pipelines, and underground wells.

The installment of on-site water storage tanks as a preparedness measure was described in six studies, among which more than half were conducted in Japan in the aftermath of the 2011 Great East Japan Earthquake disaster (29, 34, 39, 47). Results from one study conducted in 134 disaster-based hospitals in the capital area of Japan show an average water tank capacity of 8.32 l/m2, providing hospitals with water for approximately one day, if planned accordingly, in case of an emergency (29). In a study conducted in Miyagi Prefecture, the majority of the 14 disaster-based hospitals had water storage capacity of less than a day, highlighting the need for a clear water allocation plan (34). In large Japanese university facilities (650–800 beds), a large water tank of 700 m3 and dual water tanks of 160 m3 each, respectively, would allow to supply for approximately one day based on an approximate daily consumption of 500–600 m3 on peak weekdays (39, 47). Results from a survey conducted in 54 major health care facilities (i.e., hospitals, health centers and health posts) in the Greek Islands show that more than two third (70%) of facilities had a backup water tank available, among which half of the hospitals reported having reserve of water for three or more days (15). In the United States, as part of a hurricane-protection master plan, a children's hospital in New Orleans has installed 4 water tanks of 15 m3 each to provide water in the event of an emergency (40).

The implementation of external hook-ups for permanent water hoses and/or piping in disaster-based hospitals and academic medical centers was described in four studies as a measure to prepare for water outages or impairments (21, 27–30). After Hurricane Katrina, a large medical center (700 beds) in the United States installed external hook-up for emergency water supply in its new buildings as well as a back-up groundwater well to supply air conditioning chillers (HVAC) (21). Similarly, in the aftermath of a chemical spill in West Virginia, two hospitals in the United States proceeded to the installation of water-intake site where tanker trucks would be able to deliver water in case of emergency (27). To prepare for earthquakes, one disaster-based hospital in Japan designed internal mixed water systems with double water pipelines for the hemodialysis center: one main line supplied with tap water and well water and one back-up pipeline connecting the well to the hemodialysis center (28). Similar dual systems exist elsewhere, for example, in Malaysia internal water supply systems in hospitals were generally divided into two sub-systems for facility usage (e.g., chillers and air-conditioning systems, medical equipment) and staff or patient usage (e.g., dialysis services, laboratories, surgery wards and sanitation facilities) (30). Results from a survey conducted in 134 disaster base hospitals in Japan show that the number of hospitals that use well water, rainwater, and reclaimed water systems have increased recently, with a respective adoption rate of 30, 17, and 30% (29).

##### Emergency water supply plans and tools

Guidelines and tools to assess and improve the state of emergency preparedness for water supply outages or impairment were explicitly discussed in one quarter of the studies included in the literature review (Table 5). The Hospital Safety Index (HSI) and the American Emergency Water Supply Planning Guide for Hospitals and Healthcare Facilities (EWSP) were found to be the most used or referred guidelines for risk assessment and preparedness planning for water outages or impairments. Designed by the World Health Organization (WHO) and the Pan-American Health Organization (PAHO), the HSI has been used to assess hospital safety and subject of several studies worldwide (32). Without surprise, the HSI was identified as the most used tool to assess the state of health care facilities' preparedness in studies identified through this review. The application of the HSI varied according to the type of facility, e.g., hospitals, primary health care centers and health posts, hospital capacity i.e., ranging from 18 to 980 beds, in diverse locations e.g., Greece, Indonesia, Iran, Serbia. It was used to assess the health care facilities' non-structural safety, which included water supply systems, locations of water tanks, water quality control, sanitation systems, heating, ventilation, HVAC, and/or hot water systems. In Greece, Iran and Indonesia, the HSI was also used to formulate recommendations to improve water supply safety (15, 26, 33), whereas in Serbia the usefulness of the HSI for safety assessment of a primary health care center was further evaluated (32).

**Table 5:** Preparedness tools for emergency water supply in health care facilities in the literature (*N* = 10/39).

**Tools**	**Use**	**Number of studies**	**References**
American EWSP	Presentation, reference and critics	5	(18, 21, 42, 44, 47)
HSI	Assessment, recommendations and critics	4	(15, 26, 32, 33)
South African water supply risk management and response plan	Assessment and recommendations	1	(18)
Disaster action plan for critical engineering	Assessment	1	(30)
BIA	Assessment	1	(47)
No tool mentioned and used	NA^a^	29	All other papers from the literature review

^*a*^NA, non-applicable.

The EWSP is mainly presented as a reference tool for emergency preparedness. The EWSP was designed by the Centers for Disease Control and Prevention (CDC) and American Water Works Association (AWWA). Studies conducted in the United States highlighted the value of the EWSP standards to help health care facilities prepare, respond, and recover from a total or partial interruption of the facility's normal water supply (21, 42, 44). Studies from outside the United States, however, bring up a more skeptical perspective on the relevance of the EWSP (18, 47). For example, a study conducted in Japan broaches that the EWSP does not assign priority to operations or estimate daily water consumption in a fully operating hospital (47). In South Africa, the EWSP was further used together with the prevention, preparedness, response, and recovery model (PPRR) to develop a regional water supply risk management and response plan to reflect the institution's specific requirements (18).

A set of alternative assessment tools to prepare for emergency water supply in health care facilities were identified (18, 30, 47). One Japanese study used a business impact analysis (BIA) methodology applied to emergency water supply to analyze water use and prioritize water consumption in each department of a large hospital (653 beds) and the options for securing water in an emergency. The BIA aimed to optimize ways to use and conserve water and increases of hospital's abilities to manage disruption in the water supply (47). Similarly, a risk management plan for the continuous supply of water of hospitals was designed for hospitals in the Western Cape Province in South Africa (18). Reflecting institution's specific requirements, the risk management plan was adapted as general guidelines for the Western Cape Department of Health to assist in developing risk management and response plans for all its health care facilities. Lastly, a comprehensive hospital disaster action plan to face water and power supply was developed for five Malaysian hospitals using the Hazard Identification, Risk Assessment and Risk Control (HIRARC) guidelines for the risk analysis process as well as the UNISDR guidance note on Emergency and Disaster Preparedness for Health Facilities for the action plan (30).

##### Emergency preparedness and recommendations

Studies included in this literature review also provided recommendations on emergency water supply and waste water management (22 studies). They detailed different stages of preparedness and the responsiveness of actors and networks during a crisis.

Multiple studies have formulated recommendations on different stages to prepare health care facilities for a water outage or impairment (7, 21, 28, 34, 36, 47, 52). In addition to the general benefits of saving water, reducing water demand was identified as an important resource in case of emergency (18, 21). Health care facilities should enhance conservation practices and adopt technologies that are less water dependent in normal times. Integration of such principles will facilitate implementation of future contingent conservation protocols and further help to identify strategies to meet residual water requirements in case of emergency (52). Having a clear understanding of the priority of operations and of the initial water demand and minimum daily water requirements is a prerequisite before selecting feasible preparedness measures (18, 20, 28, 42, 47, 52). For example, this can include an evaluation and assessment of each unit or station water use as well as of hospital processes which could be maintained or replaced by waterless alternatives during emergency settings (8).

Health care facilities, particularly health care facilities whose support is expected in a disaster event, should have a clear understanding of the initial water demand and minimal daily requirements to meet specific facility needs. This is necessary to conceiving appropriate preparatory measures, including water allocation plans and water supply alternatives (20, 21, 28, 34). Attention was also driven on wastewater management and the need to consider systematic flushing plans in the preparedness, e.g., flushing toilets with non-potable water (20).

**Figure 2:**
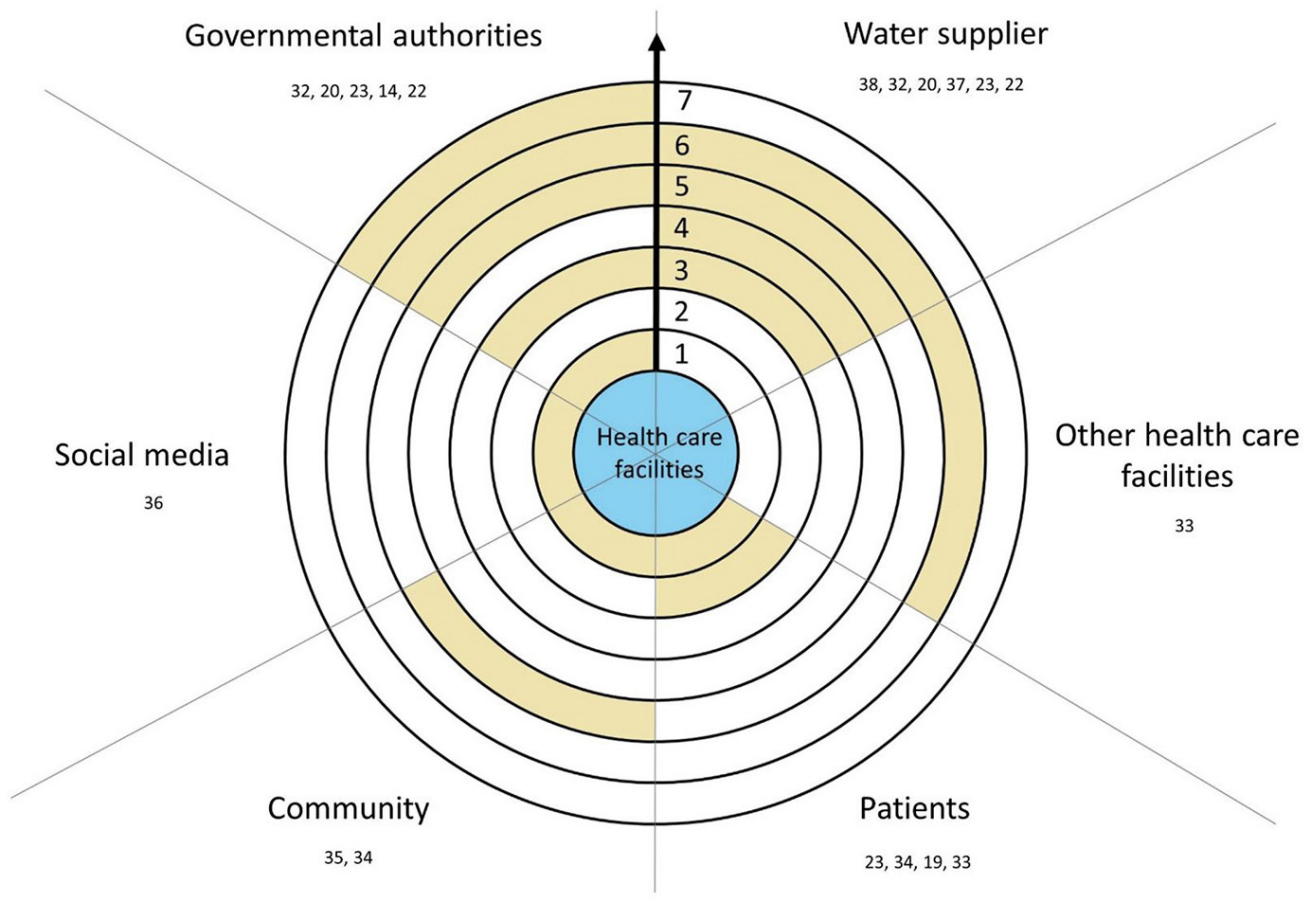
Recommended involvement of stakeholders in health care facilities preparedness. Scale: (1) Consideration, (2) Raising awareness, (3) Dialog, (4) Regular meetings, (5) Plan, (6) Agreements, (7) Legislation. Color: Yellow, Recommended level of involvement in the preparedness; White, Nothing recommended; Blue, Health care facilities. → , Progression of the formalization of networking with health care facilities.

Provision of alternative water and wastewater services should be organized with all stakeholders to aim at rapid recovery and return to baseline function (36). Stakeholders mentioned in the reviewed papers as needing to be involved in the preparedness are governmental authorities, water suppliers, other health care facilities, patients, communities and social media (Figure 2). Papers mentioning these stakeholders also discuss the different ways in which health care facilities could engage with them, from informal to formal (consideration, raising awareness, dialog, regular meetings, plans, agreements, legislation). Overall, health care facilities, governmental authorities and water suppliers were identified as foremost stakeholders for water emergency preparedness and linked through trilateral or bilateral coordination efforts. Multiple studies also identified patients as important actors to involve in the emergency preparedness phase (28, 37, 40, 43). To a lesser extent, other health care facilities which are not affected by the outage or impairment, the community in which the hospital structure is located, and social networks are also mentioned as essential to enhance networking responsiveness and communication plans (23, 37, 41).

The formalization of the cooperation into plans and/or agreements is recommended to link governmental authorities, water suppliers, other health care facilities, communities and patients with health care facilities in the preparedness and response phase (19, 25, 28, 30, 43). Adopting sustained measures to ensure availability of emergency water supply can ease thought appropriate emergency and security legislation (28). Health care facilities need to be part of the emergency water supply plan of a municipality and considered when planning the distribution of water supply and should prepare according to the emergency water supply plan and capability of the local government (20, 29). Likewise, health care facilities and water suppliers should engage one another in their respective service areas to develop effective emergency operation plans for healthcare facilities, for example through regular disaster management meetings or memorandum (25, 28, 30). After the Great East Japan Earthquake, dialysis centers of the Fukushima Prefecture for example proposed a law to the local government to guarantee them priority over a certain amount of water in case of emergency.

Health care facilities play a central role in area-wide disaster and evacuation planning and emergency preparedness should be considered as a part of the reinforcement of community resilience (23, 37). Raising awareness about the importance of water in health care facilities is recommended with water suppliers and patients, for example using social media or fact sheets (41, 43). Multiple studies have further identified the patients as an important actor to involve in the emergency preparedness phase (28, 37, 40, 43). The broader community, including function of social media, was identified as essential to enhance networking responsiveness and communication plans (23, 37, 41).

#### Emergency response

A majority of the studies included in this review were disaster case reports retrospectively assessing emergency preparedness and response in health care facilities (23 studies). Emergency response following an occurrence of water supply outage or impairment was evaluated through various operations, including emergency water and sanitation services, emergency plans as well coordination and communication measures implemented.

##### Emergency water supply and sanitation services

Overall emergency water supply was generally provided through alternative sources coming either from inside or outside the health-care facility: bottled water, water tanks, water trucks, wells, water supply treatment unit, waste water treatment unit (16 studies) (Table 6). Efficiency of those measures and actors involved in the provision of water supply was discussed disparately in the literature.

**Table 6:** Emergency response measures in the literature (*N* = 18/39).

**Implemented emergency water supply**	**Number of studies**	**References**
Bottled water	6	(17, 21, 22, 27, 31, 41)
Water tanks	6	(17, 34, 35, 39, 44, 47)
Water trucks	2	(7, 49)
Wells	5	(17, 21, 28, 34, 50)
Water supply treatment unit	1	(17)
Waste water treatment unit	1	(25)
**Emergency plans**		
Used for provisions of food, water, medications, security and physician	1	(23)
Used as a support to respond to the crisis	1	(40)
Used with modifications when necessary, including essential staffing, social media, water supplies, dialysis process, and communications	1	(41)
**Actors involved in the implementation of emergency water supply**		
Hospitals	6	(28, 31, 34, 35, 39, 47)
Private sector	6	(7, 17, 21, 35, 41, 50)
Rescue agencies (fire fighters, red cross etc.)	3	(17, 21, 44)
Governmental actors (regional/national authorities, army)	2	(49, 50)
Volunteers	1	(17)
International actors	1	(50)

Regardless of the type of emergency, e.g., earthquakes, or chemical spills, the most common and first hospital response was the provision of drinking water using bottled of water (17, 21, 22, 27, 31, 41). All studies, except one conducted in Sri Lanka, reported on disasters that happened in the United States. The provision of bottled water was eased in cases where hospitals had previously stored bottled of water on-or off sites (31, 41), otherwise they would rely on the local soft drink distributor. The use of couriers, for example to move the bottles through the hospital, was identified an important asset to facilitation emergency response. In Sri Lanka, in the first days after the 2004 Indian Ocean earthquake and tsunami, water was collected from bottling plants located in non-impacted areas and distributed in affected areas. Bottled of water was mainly used to provide drinking water to the patients, ensure basic hygiene practices (27, 31) and pursue some operations in the hospital e.g., limited food preparation (21), and irrigation of endotracheal tubes (22). After a water contamination in a municipal supply in the United States, the need for a memorandum of understanding with additional water suppliers for future emergency events was highlighted (41).

Case reports following natural hazard-induced disasters in Japan and Sri Lanka described the use of *in-situ* back-up water storage tanks previously installed as part of disaster preparedness measures in the affected health care facilities (17, 34, 35, 39, 47). Complementary or alternatively, tanker trucks were also reported as a common emergency external source to supply health care facilities (7, 34, 35, 39, 44). Water trucks can hook up directly to the hospital line, or in some cases to the back-up tank, or provide external supply. The availability of water trucks, quality of water as well as accessibility to health care facilities in affected areas were, however, identified as important challenges. For example, in Sri Lanka, first response was to deliver water from the truck in reused containers but this was later switched to bottled water because of water quality concerns (17). In the United States, in a case when no water was available from the municipal supply water trucks deliveries were arranged with a private construction company that had relationship with the hospital (35). In Japan, water trucks could supply the hospital with ~100 tons of water daily, but this was not sufficient to continue hospital operations after a few days (39). Lessons from emergency case reports show the importance of having reliable access to external sources of supply, e.g., initiated with water trucks, but also highlight the need to provide alternate supply that is connected to the hospital line after a few hours or days (7, 35, 44). In an effort to restore services, water treatment plant units or reverse osmosis treatment systems were commonly deployed by the national guard in response to emergency events that lead to water disruptions (49).

Whereas, issues related to the water supply are often discussed in case reports, few studies mentioned alternative measures for the provision of sanitation services and/or wastewater management within the health care facilities during an emergency. After Hurricane Isabel in the United States, surveyed hospitals reported the cascading effect of the loss of water supply on sanitation facilities and hygiene (52). As an alternative, after Hurricane Ike, water from the hospital's physical therapy swimming pool was used to flush the toilets. In the construction of a hospital to face COVID-19 in China, wastewater treatment was prioritized, as a result a leakproof sealed collection system with high-density polyethylene anti-seepage was installed to ensure water quality standards in discharge water (25).

Similarly, some hospitals have installed underground wells to provide backup source of water in prevention and/or response to an emergency. In Japan, hospitals with connected wells were able to quickly obtain sustained volume of water and operate under basic conditions after the Great East Japan Earthquake (28, 34). In the United States, firefighters were also involved in the response by pumping well water into three 2 000-gallon dump pools and pumping the water into the hospital through its external hook-up (21). Findings from Sri Lanka and Haiti provide a different perspective as wells were originally used as a primary source of water. In Sri Lanka, wells were heavily damaged by the earthquake and tsunami, and could not be used as an alternative source until they were later restored by pumping out saltwater (17). In this case, international responders, e.g., Thai Red Cross, and Canadian Disaster Assistance Response Team, eased deployment of temporary water treatment units to support HCF during emergency responses, and later allowed to develop permanent water treatment facilities. In Haiti, the local authorities organized the collection and transportation of water from deep boreholes in the capital Port-au-Prince to priority facilities such as hospitals. The involvement of the WASH Cluster in Haiti allowed for water quality testing and chlorination to be performed before distribution to health care facilities (53).

##### Emergency plans and coordination

Appropriate response to emergency events highly depends on quickly implemented response measures, and this can be facilitated through existing emergency plans and coordination mechanisms (11 studies).

Nevertheless, the use of emergency plans was infrequently reported in case report studies. Overall, cases that reported the use of emergency plans referred to events that took place in the United States. For example, large hospitals in New Orleans developed extensive hurricane-protection master plans (23, 40). The state of preparedness as well as management of hospital operations allowed for quick implementation of the disaster plan days before and after Hurricane Katrina. Similarly, a large hospital system provided a quick response to a “do not drink, do not boil” advisory, using and adapting a designated emergency operations plan (41). The inability to communicate during the emergency was, however, cited as an important limitation to the implementation of emergency plans (23).

Considering that multiple stakeholders and actors are involved during a water emergency, integrating coordinating efforts and breakdown structures and roles is necessary. Emergency operation centers are of critical importance to coordinate operations and management of the infrastructure during and after a water crisis (31). In a Chinese COVID-19 temporary field hospital, an online communication platform, gathering different expert groups, was implemented to provide online technical guidance for the management of water and wastewater (25). Lack of coordination can have significant impact, particularly on the ability to respond quickly to an emergency. For example, during Hurricane Katrina, the last hospital to evacuate newborns was the one that couldn't rely on immediate assistance from contacts in other states or its parent organization (40). There is a need to integrate all stakeholders in emergency plans. In response to a cholera epidemic, the Ministry of Health in Zambia activated a national emergency operations center, using an incident management system to collaborate with other government ministries and partner organizations, e.g., CDC, Africa CDC, UNICEF, WHO, Zambia Red Cross, Médecins Sans Frontières, and others (46). Results from case studies conducted in Zambia, Haiti and Sri Lanka show that involvement of International Disaster Relief agencies can further facilitate the provision of services from a humanitarian to development perspective, e.g., temporary water treatment plants replace by permanent infrastructure.

When water is not readily available and/or emergency plans fail, countermeasures must be implemented by health care facilities. Damage to the water supply system will mainly influence the decision of health care facilities to relocate patients or evacuate as a response measure. The decision of a facility to evacuate will be based on its ability to ensure safety and meet the needs of the patients. Case studies conducted in the context of high impact natural hazard-induced disasters, e.g., hurricane, earthquake, and floods, shows that evacuation was necessary (22, 34, 36, 40, 45, 48). In some cases, total evacuation was completed in < 36 h (22, 40). Various factors including viability of resources outside the hospital and damage to other critical infrastructure, e.g., exit routes, and transportation available, will influence response. A combination of internal and external coordination measures, including preparedness training, communications, evacuation of patients and involvement of volunteers, were identified as important factors to address emergency response (36). Various studies highlighted the importance of establishing an effective communication system. Use of informal channels such as social media can play an important role during a crisis, but, in some cases, fast media communication was also identified as a drawback (36), e.g., during a crisis in the United States, media information was shared more quickly than the updates from the hospital's emergency management team which caused confusion and interference with internal policies (41).

##### Recovery planning

Although it is important to look forward to the reconstruction phase and restoration of services (36) the preparation of recovery plans is rarely discussed in the literature. Five case reports from Japan and the United States, however, reconsidered emergency preparedness and self-sufficiency of health care facilities to rethink failure (21, 27, 31, 34, 41). Regardless of the type of emergency that was faced by health care facilities, i.e., chemical spill, interruption of service or massive damage to water supply, permanent measures were considered and/or implemented to reverse the impacts on services and prevent failures. Management group discussions and after-action review led, among other things, to changes in hospital policies and restructuration of infrastructure (31). Most health care facilities used engineering-based techniques to secure both existing and new infrastructure. For example, after a chemical spill in the United States, the affected hospital included a centralized water-shut-off mechanism and a water-intake site where tanker trucks could deliver water to existing renovation plans (27). Similarly, after Hurricane Floyd, a new non-permitted well with stand along water treatment plant as well as emergency water supply hooks up were installed in a large academic medical center in North Carolina, United States. Additionally, water-cooled systems of the same hospital complex were converted to air cooled to ensure essential functions (21). More extensively, the Ministry in Japan enhanced the earthquake resistance of disaster base hospitals, and as of 2012, 73% of disaster base hospitals and critical care centers were considered earthquake proof (34).

## Discussion

This research aims at assessing existing preparedness and response structures and mechanisms to support water and sanitation services in health care facilities in the context of emergency settings and low-resource contexts. Studies were found in emergency settings, either in high-, middle- and low-income countries. The following section discuss the emergency preparedness and response measures, looking at if they were concretely implemented or part of recommendations, as well as where they were located. The lack of recovery measures and analysis of cascading impacts in the literature is also questioned. Gap in research on actor collaboration and cooperation is highlighted, particularly with regard to the role of citizens and patients.

Overall, the lack of available literature from low-resource contexts which are frequently dealing with impairments of water and sanitation services is concerning, as much can be learned from such contexts for emergencies in locations with less frequent disruptions. Only very few case studies were found in such contexts, while the majority of studies comes from the United States and Japan. Both countries are notably regularly affected by disaster events, mostly induced by natural-induced disasters and much can be learned from their mechanisms. Nevertheless, this poses a significant gap and leaves a somewhat one-sided picture.

Multiple sources of water can be used to supply health care facilities during an emergency. Given the urgency to act in case of water outages or impairments in health care facilities it is surprising that only 10 studies were found on emergency water supply preparedness measures. The key measures found, namely back-up water storage tanks, additional pipelines, and underground wells, however, seem universally applicable, and not limited to specific locations. Besides these sources few alternatives are mentioned, raising questions on how to improve existing technologies, and their uptake, but also the supplementation by other creative measures that are adapted to specific contexts, such as supply by water trucks (7, 34, 35, 39, 44). The availability of emergency *in-situ* water treatment is also important to provide safe water, but this was overlooked in most cases.

A potential reason for the lack of emergency supply measures–whether theoretically possible or already implemented–could be a related lack in water supply plans and tools. Comparably few health care facilities discussed in literature comprised of such plans, with the most frequently debated one being developed by international (e.g., WHO) or US American Organizations. Likewise, the case studies from the United States, that seem to have a well-referenced water supply plan, do not regularly mention its uptake. Few studies mentioned national tools from other countries, again Japan was one of the cases described (18, 47). This suggests, unsurprisingly, that concrete threats lead to more planning, such as the extreme earthquake risk in Japan (28, 29, 34, 39, 45, 47), hurricanes in the US (21–23, 35, 39, 41, 43, 52) or drought risk in South Africa (18) where the case studies with concrete policies and preparedness measures came from. Overall, the lack of plans mentioned in literature is concerning and has the potential to impede better preparedness. It remains to be analyzed whether this is because such plans are not considered worthy of research in the context of health care facilities. Even if they are described in detail in the gray literature, a gap remains.

The most significant and concrete on-site preparedness measures were found in Japan, where disaster preparedness has been a prioritized national agenda (54). This can be explained by the high risk of earthquakes and the associated preparation for such events, also in health care. All health care facilities analyzed in the review are comparably big and would be important supply points in case of a disaster. Japan also has a particular status for Disaster Base Hospitals (28), coming with legal obligations regarding water supply in times of emergency, namely tray water tanks of appropriate capacity and wells. Nothing comparable could be identified, although across papers a slight correlation between the facility size and overall preparedness could be found, in general small facilities tend to be less prepared than bigger ones. The reasons for this may be the lower availability of financial and technical resources, but also the lower staffing level, which usually does not provide for a specialized staff member for the topic. The absence of guiding documents and legal requirements may also play a major role for lack of preparedness, since especially for smaller facilities, own planning is probably not within the realm of possibility. However, it is precisely in these facilities that a large proportion of patients are cared for on a decentralized basis in case of a larger emergency.

Scientific literature that focus on recovery mostly discussed technical aspects, while organizational ones are largely absent. Whether this is due to the non-existence of such measures in reality or to a lack of research or a lack of adequate analytical tools to assess their efficacy remains unknown. However, it is reasonable to think that this is also due to a one-sided focus on the technical solvability of the water supply. The most commonly used response for provision of drinking water seems to be the use of bottled of water, raising the question of how long this might sustain functionality of the respective health-care facility that would usually have more critical functions needing water in larger amounts. Evidence from this review shows that, in most cases, evacuation was necessary when water couldn't be further provided (22, 34, 36, 40, 45, 48). The use of bottled of water is often necessary in the first hours after a disaster but should not be considered a viable option for long-term water supply, especially for other purposes than drinking. Wells seemed to be an interesting alternative to rapidly provide emergency water supply, particularly when connected directly to the main line through a back-up pipeline, again Japan is an example where comprehensive plans including the construction of wells to improve response (28, 29, 34). Water trucks were mentioned as an alternative supply source in different countries and contexts, however, papers usually fall short on assessing their viability in case of a larger crisis that might affect access to the respective facility, for instance if roads are blocked or flooded. Moreover, reliability of water trucks would also depend on existing infrastructure to distribute water following the disaster, e.g., external hook-up for emergency water supply and water tanks.

Overall, cascading impacts of any crisis on health care facilities were little discussed throughout preparedness, response and recovery. Sanitation during a crisis is looked at even less. This is concerning as for example a paper on US hospitals reported on hygiene problems due to lack of sanitation (22) which can easily disrupt functionality of health care facilities and delay the resumption of operation. Evidence from the field of WASH shows an international consensus on the impact of lack of access to water supply on the provision of sanitation and hygiene services, including in health care facilities (2, 55–57). Multiple papers from low-resource context, e.g., lower economies, that were initially retrieved from this systematic review were excluded as their main focus were overall WASH conditions and coverage of access. Given that WASH as overarching topic is very prominent the lack of research publications addressing it comprehensively for health care infrastructure is even more obvious. A potential reason is that WASH and basic service levels are usually associated with crisis or emergencies in low-resource contexts, where focus is often directed toward lacking services, and much less in countries or regions with high standards and security of supply (8). This is an opportunity missed to learn from low-resource contexts, overcoming challenges that relate to water supply, e.g., water intermittency in municipal water supply and wastewater management, which are well-known but rather scarce in research literature.

Another key finding of the analysis is the lack of recovery planning, which is mentioned in few publications only. However, it is exactly in this phase where learning from lack of preparedness or mistakes in recovery can be addressed and improved. Case studies found are from the United States (21, 27, 31) and Japan (34, 39) again, and link both, organizational and technical aspects. Concrete cases describe interventions to improve technical setups based on learning from failure. This remarkable step may also be the reason why so little is read about it-it requires a high degree of critical faculties and openness to make these mistakes in preparedness and recovery public. Likewise, there is a lack of literature on actor cooperation during the different phases of emergency planning, which could blind to its potential. There is a need to further explore the interactions, as well as collaboration and involvement of all actors. Also, perplexing was how little information related to cooperation or collaboration between different actors to prepare for crisis situations regarding water supply and sanitation in health care facilities was given in literature, although they are discussed in crisis response. The importance of considering water demands of hospitals and other health care facilities as part of a larger local and global disaster management has been emphasized by several authors across the board. However, the analysis did not yield any preparedness measures, i.e., formalizing plans or agreements between actors implementing regular meetings or installing dialogue formats and exchanges to raise awareness. Actors that should be involved in both preparedness and response according to literature are numerous and include state or private actors, as well as citizens, volunteers or patients with specific needs. Except for water suppliers when they are private enterprises, the private sector is not taken into consideration in the implemented preparedness measures, but only in response. Their involvement in the response was mentioned in Haiti, the United States, and Sri Lanka case studies (17, 35, 53).

This systematic review suggests that citizens and patients as actor groups across phases are hugely underrepresented in research, almost forgotten issue. Not much data is available on them, despite their direct vulnerability to water outages and their key role for both, preparedness and response. This raises the critical question of whether literature on water and health infrastructure tends to be more traditionalist, concerned with public actors and technical solutions, but blind to social and organizational concerns. In the event of a crisis, however, such aspects are of fundamental importance, as key actors responsible for functioning health care facilities can quickly become affected people who are themselves dependent on health care and who want to ensure the wellbeing of their families (39). Communication and awareness raising about the risks water supply outages for and across different actor groups is likewise hardly addressed beyond stressing the need.

Finally, a discrepancy was found between proposed preparedness as well as response measures and those implemented e.g., not many plans and communication with external stakeholders are mentioned in the actual preparedness, while the need is stressed all over. Another example is evacuation plans that do exist but that are not mentioned in preparedness, making actual preparedness partly difficult to assess. Many case studies reported the need to specifically improve water supply preparation, but the assessment of hospital preparedness is often limited to its overall evaluation, e.g., using hospital safety index (15, 26, 33). Health care facilities with reported preparedness and response mechanisms are mostly among the larger ones described, raising the question if smaller facilities are really less prepared or simply less researched and less often rescued during emergencies. There is a demand for studies covering the different phases, which would allow for assessing if a health-care facility was prepared for a specific event or rather for others, and how far preparedness helped in recovery.

One limitation of this study is that scientific literature might not provide a comprehensive overview on all preparedness and recovery plans, which might be covered more in gray literature or even be unpublished, depending on the country, if, for example, their publication is judged not to be conducive to the reputation of a private health care facility or simply its security. In other cases, health care facilities might have evacuation or other emergency plans which are not for water supply and sanitation problems only, but which might be activated in case of an outage. Another limitation is the underrepresentation of healthcare facilities other than hospitals and dialysis centers in literature. With respect to analysis, it was decided to only select and analyze such tools that were further described at least to a minimum.

## Conclusions

This systematic review highlights the importance of water supply emergency preparedness in healthcare facilities, and lack of evidence on existing structures and mitigation mechanisms. In the context of rising climate change, health care facilities are, more than ever, vulnerable to natural hazard-induced disasters and ever-increasing fragilities. All involved actors, including particularly health care facilities, water suppliers and governmental authorities, must ensure that basic WASH services can be provided and can rapidly recover in the event of an emergency, and this is particularly important for reference hospitals, including health care facilities whose support is expected in the event of a disaster, providing first emergency response. This study yields multiple insights for future research on the provision of emergency water and sanitation services. Combining organizational and technical aspects, and intersecting theory and practice will be necessary to address existing gaps.

Future research should focus on identifying strategies to enhance infrastructure resilience, both through improving existing infrastructure and implementing new technologies. In addition to research on more technically oriented aspects, research is also needed on the appropriate identification, involvement and capacitation of all relevant stakeholders. Increasing capacity response and minimizing adverse effects on critical health infrastructure is key to maintaining public health and providing primary care.

## Data availability statement

The original contributions presented in the study are included in the article/Supplementary material, further inquiries can be directed to the corresponding author.

## Author contributions

AC, SvdH, and SS contributed to conception and design of the study. SvdH and AC conducted the literature analysis and wrote the first draft of the manuscript. SvdH, AM, and AC compiled tables and figures. SS authored sections of the manuscript. All authors contributed to manuscript revision, editing, read, and approved the submitted version.

## Funding

Authors received financial support from the German Federal Ministry of Education and Research (BMBF) under its Green Talents Programme and the Natural Sciences and Engineering Research Council of Canada. Parts of the article are based on the funding of the research project NOWATER: Emergency preparedness planning for water supply and sanitation in health care facilities–organizational and technical solution strategies to increase resilience, financed by the BMBF (Grant Number: 13N15282).

## Conflict of interest

The authors declare that the research was conducted in the absence of any commercial or financial relationships that could be construed as a potential conflict of interest.

## Publisher's note

All claims expressed in this article are solely those of the authors and do not necessarily represent those of their affiliated organizations, or those of the publisher, the editors and the reviewers. Any product that may be evaluated in this article, or claim that may be made by its manufacturer, is not guaranteed or endorsed by the publisher.

## Supplementary material

Supplementary Table 1 Search strategy for Medline and Web of Science database.
